# A simulation-based network analysis of intervention targets for comorbid symptoms of depression and anxiety in Chinese healthcare workers in the post-dynamic zero-COVID policy era

**DOI:** 10.1186/s12888-025-06931-z

**Published:** 2025-05-06

**Authors:** Chao Zhang, Ruyong Li, Wei Zhang, Yanqiang Tao, Xiangping Liu, Yichao Lv

**Affiliations:** 1 School of Education Science, Shanxi Normal University, Taiyuan, China; 2 Institute of Applied Psychology, Shanxi Normal University, Taiyuan, China; 3 Faculty of Psychology, Beijing Normal University, Beijing, China; 4 Beijing Key Laboratory of Applied Experimental Psychology, National Demonstration Center for Experimental Psychology Education, Beijing, China

**Keywords:** Chinese healthcare workers, Anxiety, Depression, Comorbidity, Network analysis, NodeIdentifyR algorithm

## Abstract

**Background:**

After the official end of the dynamic zero-COVID policy in China, healthcare workers continued to heavy workloads and psychological stress. In this new phase, concerns related to work and family, rather than infection, may have become new sources of psychological issues such as depression and anxiety among healthcare workers, leading to new patterns of comorbidity. However, few studies have addressed these issues. To fill this gap, this study used network analysis to examine new features and mechanisms of comorbidity between depression and anxiety symptoms, and simulated symptom-specific interventions to identify effective targets for intervention.

**Methods:**

A total of 708 Chinese healthcare workers (71.2% females; Age: *M* = 37.55, *SD* = 9.37) were recruited and completed the Patient Health Questionnaire-9 (PHQ-9) and Generalized Anxiety Disorder-7 (GAD-7). This study first calculated the incidence rates of anxiety, depression, and their comorbidity, and then constructed the comorbid Ising network. Central and bridge symptoms were identified with expected influence (EI) and bridge EI, respectively. The *NodeIdentifyR* algorithm (NIRA) was then used to simulate interventions within the network, examining the effects of alleviating or aggravating specific symptoms on the network’s severity.

**Results:**

48.2% of Chinese healthcare workers reported experiencing depression (19.8%), anxiety (11.7%), or both (16.2%). In the anxiety-depression network, “guilt” and “appetite changes” were identified as the central symptoms, and “guilt” and “excessive worry” were identified as the bridge symptoms. Simulated interventions suggested that alleviating “Anhedonia” can the most reduce the overall severity of the network, while aggravating “guilt” can the most increase the overall severity. These two symptoms were considered the key target for treatment and prevention, respectively.

**Conclusions:**

Chinese healthcare workers still face high risk of depression, anxiety, and comorbidity in the post-dynamic zero-COVID policy era. Our findings highlight the key roles of guilt, appetite changes, and excessive worry in the network of depression and anxiety symptoms. Future research should apply the results of the simulated interventions, develop intervention strategies targeting anhedonia, and focus on preventing guilt to improve the healthcare workers’ mental health.

**Trial registration:**

Not applicable.

**Supplementary Information:**

The online version contains supplementary material available at 10.1186/s12888-025-06931-z.

## Introduction

As the COVID-19 fatality rate declined, vaccination coverage increased, and practical experience in pandemic management accumulated, China released 10 new measures in December 2022 to optimize its COVID-19 response, representing the end of the dynamic zero-COVID policy [1, 2]. These measures included lifting all forms of temporary lockdowns, allowing asymptomatic carriers and mild cases to quarantine at home, and requiring medical institutions to treat patients without refusal. However, China’s fight against COVID-19 was far from over. In the year following the policy shift, the public health system continued to face immense pressure from emerging threats and ongoing challenges. COVID-19 subvariants and seasonal flu caused intermittent low-level outbreaks, while rising long-COVID cases further burdened the healthcare system [3–5]. For healthcare workers already exhausted during pandemic, the policy shift did not necessarily translate into relief. They often struggled to adapt to the new phase due to the persistent heavy workloads and psychological stress [6]. In this new phase, concerns related to work and family-rather than infection-may have become primary sources of psychological issues such as depression, anxiety, and insomnia, leading to new patterns of comorbidity. However, few studies have focused on these issues. Therefore, this study aims to use network analysis to explore the comorbidity of depression and anxiety among healthcare workers in this new phase, as well as the simulated intervention analysis to suggest the potential intervention plans [7, 8].

Anxiety and depression are two common mental health issues that were often comorbid among healthcare workers during the pandemic [9]. Several studies conducted between 2020 and 2021 used network analysis to explore the mechanisms behind this comorbidity. Research on nurses and clinicians showed that “*trouble relaxing*” and “*uncontrollable worry*” were common central symptoms in the anxiety-depression networks [10, 11]. Due to strict quarantine policies, healthcare workers had fewer opportunities for outdoor activities to relieve stress, and were often concerned about the risk of infection for themselves and their families [10–12]. In addition, common bridge symptoms, such as “*sad mood*”, “*irritability*”, “*feeling afraid*”, and “restlessness”, reflected their dysphoria for both physical and mental discomforts, as well as the fear, frustration, and despair arising from the risks of infection and pandemic-related challenges [10, 11, 13]. Recently, Chen et al. investigated the comorbidity network of depression and anxiety among Chinese mental health professionals in public hospitals immediately after the end of the dynamic zero-COVID policy [14]. In their study, symptoms such as “*restlessness*”, “*fatigue*”, and “*feeling afraid*” were identified as the central symptoms, while “*guilt*”, “*restlessness*” and “*motor disturbance*” were identified as the bridge symptoms. Chen et al. suggested that these symptoms reflected the new challenges faced by healthcare workers during this period, such as concerns about personal and family infections, increased workloads, profound changes in work and lifestyle, and uncertainty about the future [14]. More importantly, these short-term challenges raised concerns about potential long-term impacts on healthcare workers’ mental health. This brings up key questions: Will the mental health of healthcare workers continue to evolve months or even a year after the policy ended? Will the mechanisms and explanations for comorbidity also change?

Since the end of the dynamic zero-COVID policy, the sources of anxiety and depression among healthcare workers may have shifted. While the fear of infection has decreased, healthcare workers continue to face other public health crises such as seasonal flu [5] and the long-term physical and mental sequelae of COVID-19 [15]. Studies have shown that some COVID-19 patients continue to experience symptoms, such as fatigue, weakness, cough, chest tightness, headache, cognitive decline, and psychological issues (e.g., depression, anxiety, and insomnia), for months after infection [3, 4]. These ongoing issues have further strained healthcare resources, putting additional pressure on healthcare workers. According to China’s National Health Commission (NHC), the total number of hospital admissions across the country reached 302 million in 2023, an increase of 55.01 million compared to the previous year [16]. Additionally, during the transition from crisis management to routine work, healthcare workers may feel confused, anxious, and uncertain [14]. The blurred boundaries between work and personal life make it difficult for healthcare workers to balance their responsibilities [17]. Given these challenges, it is necessary to reassess the anxiety-depression network among healthcare workers and identify central and bridge symptoms during this period. Although these symptoms are often considered targets of intervention, it remains unclear whether interventions aimed at these symptoms are effective [7]. One potential solution is the *NodeIdentifyR* algorithm (NIRA), based on the Ising network, which can simulate symptom-specific intervention [7].

The NIRA excels in simulating the changes in each symptom to generate projected networks and visualizing the magnitude of network changes [7]. The symptoms that most alleviate and aggravate the overall depressive and anxiety symptoms can be identified as the optimal prevention and intervention targets, respectively. For instance, a study using NIRA on the adolescent anxiety-depression network found that alleviating the symptom of “tension” maximized the reduction of network activation, while aggravating the symptom of “sadness” produced the most significant expected increase in network activation [18]. In this study, we would use the NIRA simulations to determine alleviating or aggravating depressive and anxiety symptoms in Chinese healthcare workers, providing targets for intervention.

To the best of our knowledge, no study has yet explored the comorbidity network of depression and anxiety among healthcare workers and developed intervention plans based on simulated intervention, especially after the dynamic zero-COVID policy ended. The gap in research motivated this study. In sum, this study aims to: 1) calculate the latest incidence rates of anxiety, depression, and their comorbidity; 2) model the Ising network of anxiety and depression and identify central and bridging symptoms; and 3) use NIRA to simulate clinical symptom-specific interventions to identify effective treatment and prevention targets.

## Method

### Participants

The study was performed in accordance with the Declaration of Helsinki and approved by the ethics committee of xxxxxxxx University (Reference number: 2022****0137). This cross-sectional study investigated the mental health status of healthcare workers from October 1 to 30, 2023, nearly one year after the end of China’s dynamic Zero-COVID policy. The study was conducted in Linfen, Shanxi Province, China. Participants were recruited from public hospitals in counties and cities, as well as health centers in rural townships, using multi-stage stratified sampling. The inclusion criteria were: (1) participants aged over 18 and of Chinese; (2) healthcare workers, including doctors, nurses, and nonmedical health care workers (e.g., allied health workers, technicians, administrators); and (3) willingness to participate in the study. A total of 708 healthcare workers (504 females, 71.2%; *M*_age_ = 37.55, *SD*_age_ = 9.37) voluntarily participated and completed the full online survey via Wenjuanxing (https://www.wjx.cn). Table S1 showed the additional demographic characteristics including marital status and annual income.

### Procedures and measures

After signing the electronic consent form, participants were asked to first provide basic demographic information (e.g., gender, age, marriage, and income per year) and then complete the assessment, including Generalized Anxiety Disorder 7 (GAD-7) and Patient Health Questionnaire (PHQ-9).

The PHQ-9 and GAD-7, both widely used in the Chinese samples [19, 20], were used to assess depression and anxiety [21, 22]. Participants rated the frequency of anxiety and depression symptoms over the past week on a 4-point Likert scale (0 = *not at all*, 3 = *almost every day*). Higher scores indicated more severe anxiety or depression. A cutoff score of five was used to screen for depression and anxiety symptoms. In this study, the Cronbach’s alpha for PHQ-9 and GAD-7 was 0.90 and 0.87, respectively.

### Statistical analysis

All analyses were performed using R software [23]. Descriptive statistics were first performed for original continuous total scores of the GAD-7 and PHQ-9, as well as the scores for each symptom. We also examined the prevalence rates of anxiety, depression, and their comorbidity, as well as the prevalence rates of each symptom. Then, we conducted the following analyses.

### Estimate network structure and centrality

Based on the continuous scores, binary scores for each symptom were calculated (absent: 0, score = 0; present: 1, score ≥ 1) and used to estimate the anxiety-depression Ising network. This network was estimated using the R package *networktools* [24], with logistic regressions conducted by iteratively regressing each symptom on all other symptoms except the symptom variable itself. The key parameters of interest were edge weights and thresholds. Edge weights, derived from the regression coefficients, represented the relationships among symptoms, while thresholds, derived from the intercepts, reflected each symptom’s tendency to manifest. Positive (negative) thresholds denote the symptom’s tendency to be activated (deactivated) if all other symptoms are absent. A larger absolute threshold value signifies a stronger tendency toward activation or deactivation. The Ising network was visualized using the *qgraph* package [25]. In the network, nodes represented symptoms, and edges represented their interrelationships. Blue edges denoted positive relationships, while red edges indicated negative relationships, with thicker edges representing stronger associations [8]. For clarity, the thresholds of each symptom were depicted individually. Notably, we also estimated a partial correlation network based on the continuous scores of each symptom and used Mantel’s statistic to assess its correlation with the Ising network. This helped us examine whether binary data could similarly capture symptom relationships [26]. A high correlation between the two network matrices would suggest that binarizing the data did not affect the network’s sensitivity.

The Expected Influence (EI) and bridge EI centrality indices for each node were estimated using the *centralityPlot* function [27]. Here, EI assessed the influence or significance of each symptom on the anxiety-depression network, and bridge EI assessed the role of each symptom in linking anxiety and depression, acting as a bridge [24]. According to previous studies, we focused on symptoms with centrality values exceeding 1 [19, 28].

### Estimate network accuracy and stability

The accuracy and stability of the Ising network were assessed using the R package *bootnet* [29]. A bootstrapping test for the edges was performed to compute the 95% confidence intervals (CIs) for edge weights, with greater overlap indicating higher accuracy. The case-dropping bootstrap tests were performed to evaluate the stability of EI and bridge EI centralities, reflected by the correlation stability coefficient (CS-C). The CS-C quantifies the maximum number of cases that can be removed while still maintaining, with 95% probability, a correlation of at least 0.7 (default) between the statistics from the original network and those from the reduced dataset. A CS-C value above 0.25 is acceptable, above 0.5 is preferred, and above 0.75 is excellent [30]. In addition, we also conducted bootstrapped difference tests for edge weights and node centrality to examine their differences or uniqueness.

### Simulated alleviating and aggravating interventions of network

Simulation intervention analyses were conducted using the *NodeIdentifyR* algorithm (NIRA) within the *IsingSampler* package [7]. NIRA simulates interventions by systematically altering the threshold parameters of the Ising network. Two types of interventions are applied: alleviating and aggravating, achieved by decreasing or increasing each symptom’s threshold by two standard deviations, respectively—representing symptom improvement or worsening. To evaluate the impact of these perturbations, NIRA calculates the sum score that reflects the overall state of the dynamic network; higher sum scores indicating greater levels of psychopathology. The symptom with the largest absolute difference between pre- and post-intervention sum scores (i.e., the NIRA outcome) is considered the most efficient intervention target in the network. Alleviating interventions identify the therapeutic targets whose alleviation most effectively reduces overall symptom severity, while aggravating interventions identify the preventive targets whose aggravation most strongly increases overall severity [7].

## Results

### Descriptive statistics

**Fig. 1:**
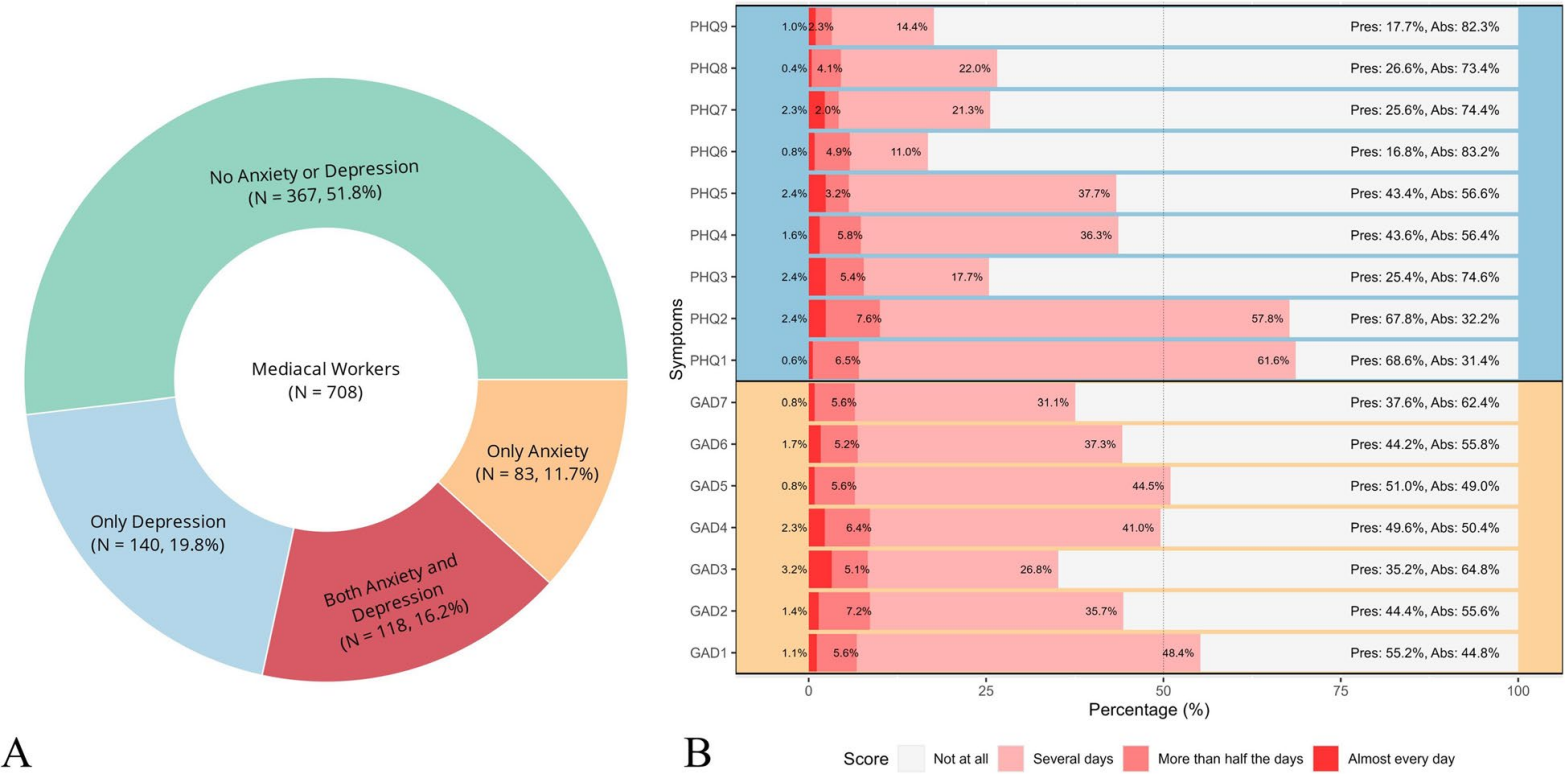
Prevalence Rates of Anxiety, Depression, and Comorbidity (**A**), and Prevalence Rate of Each Symptom (**B**). Note. the full name of each symptom in the panel B was listed in Table 1

Table 1 presented descriptive statistics for the original continuous total scores of the GAD-7 and PHQ-9, as well as the scores for each symptom. A cutoff score of > 4 on both the PHQ-9 and GAD-7 was used to indicate the presence of at least mild depressive or anxiety symptoms, respectively. As shown in Fig. 1 A, 367 healthcare workers (51.8%) had neither anxiety nor depression symptoms. Of the remaining 341 healthcare workers (48.2%), 140 (19.8%) had depression symptoms only, 83 (11.7%) reported anxiety symptoms only, and 118 (16.2%) had comorbid depressive and anxiety symptoms. Figure 1B further showed the distribution of continuous scores (0–3) for each symptom in the sample. The symptoms with prevalence rates greater than 0.5 included two depressive symptoms: PHQ1 (Anhedonia; 68.6%), PHQ2 (Depressed or sad mood; 67.8%), as well as for two symptoms of anxiety: GAD1 (Anxiousness; 55.2%) and GAD5 (Restlessness; 51.0%).

**Table 1:** Descriptive Statistics for Anxiety (GAD-7) and Depression (PHQ-9)

Symptom	Descriptive Statistics				Centrality	
	***M***	***SD***	***Skewness***	***Kurtosis***	***EI***	***Bridge EI***
**Anxiety (GAD-7)**	3.81	3.53	1.52	2.38		
GAD1: Anxiousness	**0.63**	0.64	0.78	0.70	−0.85	0.22
GAD2: Uncontrollable worry	0.54	0.69	1.14	0.96	−1.47	−0.26
GAD3: Excessive worry	0.47	0.74	1.70	2.59	−0.58	1.72
GAD4: Trouble relaxing	**0.60**	0.71	1.12	1.21	−1.00	0.17
GAD5: Restlessness	0.58	0.64	0.83	0.53	−1.13	−0.89
GAD6: Irritability	0.53	0.68	1.23	1.52	−0.32	−0.89
GAD7: Felling afraid	0.45	0.64	1.31	1.36	0.46	0.82
**Depression (PHQ-9)**	4.05	4.22	1.80	3.73		
PHQ1: Anhedonia	**0.76**	0.59	0.27	0.37	0.72	−0.89
PHQ2: Depressed or sad mood	**0.80**	0.68	0.73	1.10	−0.07	−0.89
PHQ3: Sleep difficulties	0.36	0.69	2.08	3.94	0.01	0.24
PHQ4: Fatigue	0.53	0.68	1.22	1.36	0.60	−0.35
PHQ5: Appetite changes	0.51	0.68	1.42	2.38	**1.35**	−0.89
PHQ6: Guilt	0.23	0.57	2.59	6.35	**2.41**	**2.28**
PHQ7: Concentration difficulties	0.32	0.63	2.31	5.88	−0.32	−0.89
PHQ8: Motor disturbances	0.31	0.57	1.77	2.79	0.61	−0.44
PHQ9: Suicide ideation	0.22	0.53	2.76	8.49	−0.43	0.94

Higher scores in GAD-7 and PHQ-9 are indicated in bold. Standardized EI and bridge EI were shown. Highlight EI and Bridge EI above 1 in bold

### Comorbidity network of anxiety and depression

**Fig. 2:**
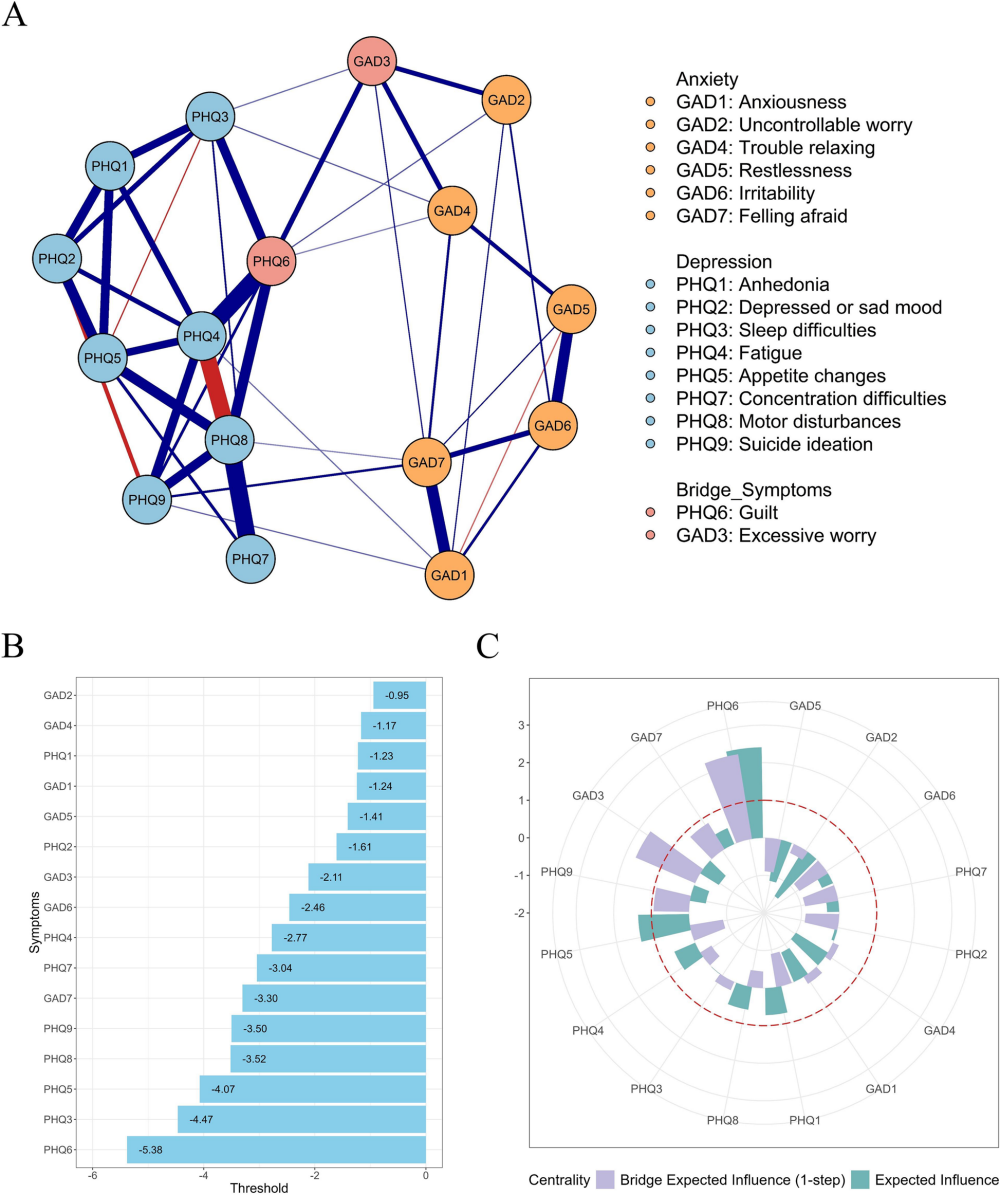
Comorbidity Network of Anxiety and Depression (**A**), Thresholds (**B**), and Standardized Centrality measures (**C**) of each symptom. Note. (A) In the network, blue/red edges represent positive/negative associations. (B) In the panel B, negative threshold reflects a tendency for the symptom to be deactivated when other symptoms are absent, with value closer to zero indicating greater likelihood of activation

As shown in Fig. 2A, depressive and anxiety symptoms formed two interconnected clusters within the network^1^ (see the edge-weight matrix and threshold parameters in Tables S2). Figure 2B indicated that all symptom had negative thresholds and thus tended to be deactivated when all other symptoms were absent. GAD2 (Uncontrollable worry) had the threshold closest to zero and was therefore the most likely to be activated, whereas PHQ6 (Guilt), with the lowest threshold, was the least likely to be activated.

As shown in Fig. 2C and Table 2, PHQ6 (Guilt; *bridge EI* = 2.28) and GAD3 (Excessive worry; *bridge EI* = 1.72) had the highest bridge EI values and were identified as the primary bridge symptoms. Among the connections between depression and anxiety, the connection between PHQ6 (Guilt) and GAD3 (Excessive worry; *edge weight* = 1.12) was the most significant. Additionally, PHQ6 (Guilt) was positively connected with GAD2 (Uncontrollable worry) and GAD4 (Trouble relaxing), while GAD3 (Excessive worry) was also positively connected with PHQ3 (Sleep difficulties). Furthermore, PHQ6 (Guilt; *EI* = 2.41) and PHQ5 (Appetite changes; *EI* = 1.35) were identified as the central symptoms with the highest EI values in the network. Besides its external connections to anxiety symptoms, PHQ6 (Guilt) also displayed strong positive internal connections with other depressive symptoms, with the strongest connection being with PHQ4 (Fatigue; *edge weight* = 3.07). Meanwhile, PHQ5 (Appetite changes) exhibited strong positive connections with five depressive symptoms (but not with any anxiety symptoms), particularly with PHQ8 (Motor disturbances; *edge weight* = 1.93).

**Table 2:** The Results of Simulated Weakening and Enhancing Interventions

Symptom	Original Sum score	Alleviating Intervention		Aggravating Intervention	
		**Sum score**	**NIRAoutcome**	**Sum score**	**NIRAoutcome**
**Anxiety (GAD-7)**					
GAD1: Anxiousness	6.17	4.90	1.27	7.43	1.26
GAD2: Uncontrollable worry	6.17	5.02	1.15	7.26	1.09
GAD3: Excessive worry	6.17	5.11	1.06	7.86	1.69
GAD4: Trouble relaxing	6.17	4.87	1.30	7.38	1.21
GAD5: Restlessness	6.17	5.10	1.07	7.38	1.21
GAD6: Irritability	6.17	4.98	1.19	7.66	1.49
GAD7: Felling afraid	6.17	5.05	1.12	8.04	1.87
**Depression (PHQ-9)**					
PHQ1: Anhedonia	6.17	4.10	**2.07**	7.21	1.04
PHQ2: Depressed or sad mood	6.17	4.34	1.83	7.33	1.16
PHQ3: Sleep difficulties	6.17	5.60	0.56	8.42	2.25
PHQ4: Fatigue	6.17	4.63	1.54	7.73	1.57
PHQ5: Appetite changes	6.17	4.70	1.47	7.88	1.71
PHQ6: Guilt	6.17	5.34	0.82	8.89	**2.72**
PHQ7: Concentration difficulties	6.17	5.71	0.46	8.05	1.88
PHQ8: Motor disturbances	6.17	5.69	0.48	8.12	1.95
PHQ9: Suicide ideation	6.17	5.71	0.46	8.24	2.07

The highest NIRA was highlighted in bold. NIRA outcome was the absolute difference between pre- and post-intervention sum scores

### Network accuracy and stability

The bootstrap analysis for network accuracy indicated that the edge-weights achieved acceptable levels of accuracy with narrow 95% CIs (see Figure S2). The case-dropping bootstrapping analysis revealed that EI (CS-C = 0.52) and bridge EI (CS-C = 0.28) indices obtained stable results (see Figure S3). Moreover, the bootstrapped difference tests for edge weights and centrality suggested that the majority of comparisons of edge weights (see Figure S4) and EIs (see Figure S5 A) were significant, suggesting that the estimates of edges and EIs are specific. In contrast, there are fewer significant differences in the comparisons of bridge EIs (see Figure S5B).

### Simulation of alleviating and aggravating interventions

**Fig. 3:**
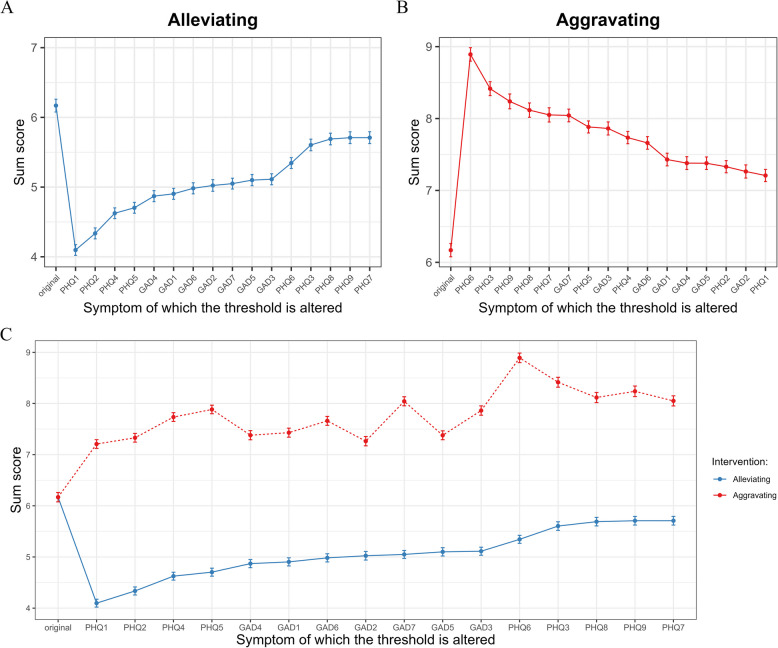
Simulation of Alleviating (**A**) and Aggravating (**B**) Interventions and Comparison (**C**). Note. Dots represent the network sum scores, while the lines represent the 95% confidence intervals. The symptoms are listed based on the size of the intervention’s effects, along with the original total score before the intervention

As shown in Fig. 3 and Table 2, different symptoms had varying projected influences on the entire network when targeted with interventions. In the alleviating interventions (Fig. 3A), alleviating PHQ1 (Anhedonia) could significantly reduce the projected sum score from 6.17 to 4.10 (*NIRA* = 2.07), which was the max decreases of overall symptom levels of the network. In the aggravating interventions (Fig. 3B), aggravating PHQ6 (Guilt) significantly increased the projected sum score from 6.17 to 8.89 (*NIRA* = 2.72), which was the max increases of overall symptom levels. Therefore, these two symptoms may be the most effective targets for treatment and prevention, respectively. Furthermore, as shown in Fig. 3C, the same node played distinct roles in spread or hinder symptom activity. For instance, symptom PHQ1 (Anhedonia) was the most suitable target for clinical interventions, as an alleviating intervention on it could furthest lower overall depressive and anxiety levels. However, it might be not suitable for prevention, since it would not have a significant impact on network even if it worsened.

**Fig. 4:**
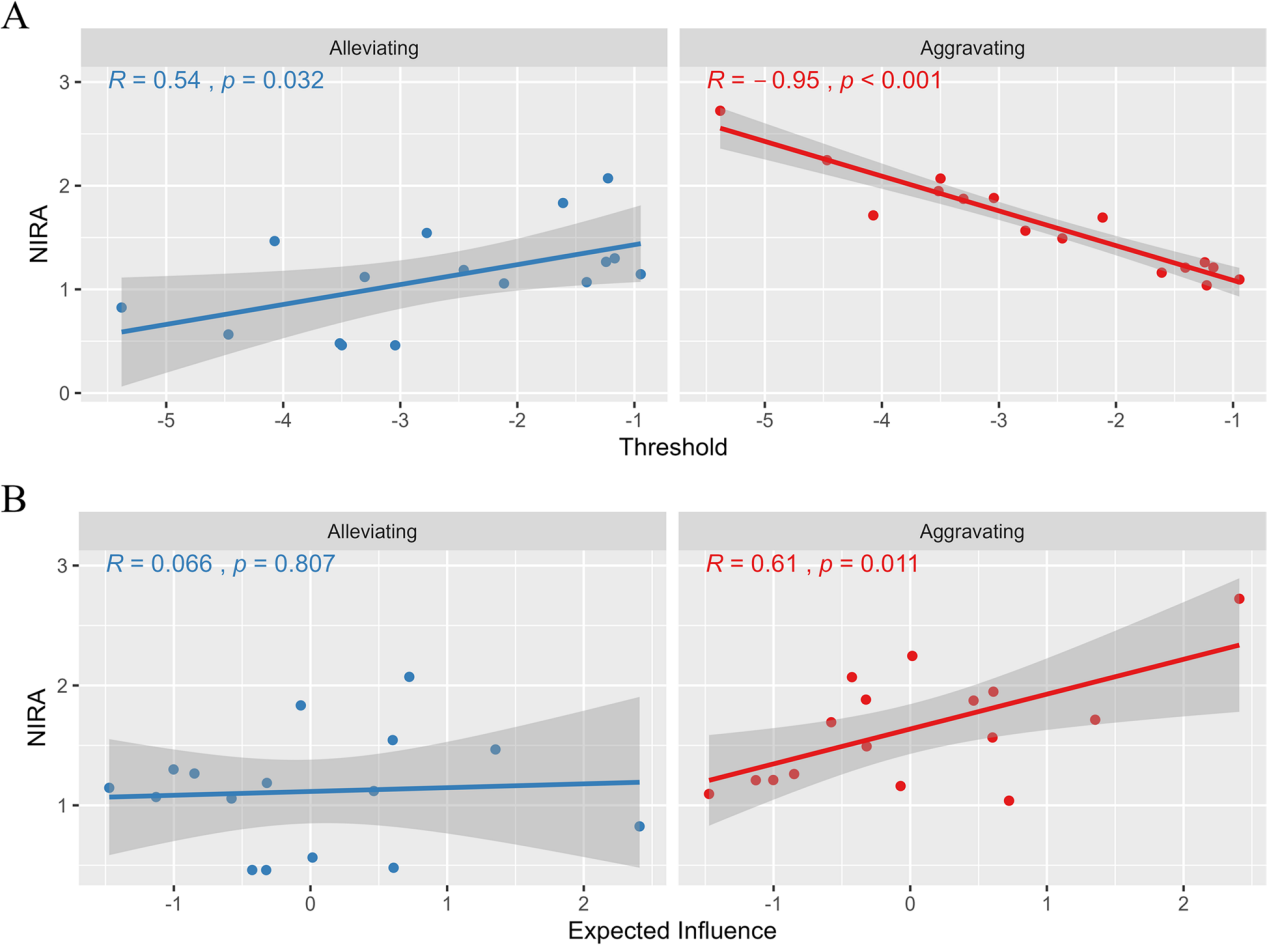
The Relations between Thresholds (**A**) and EIs (**B**) and NIRA Outcomes from Simulated Interventions. Note. The x-axis represents the magnitudes of the threshold parameters (panel A) and EI values (panel B). The y-axis displays the NIRA outcome, calculated as the absolute difference between the network’s original sum score and the sum score following each intervention. The shaded region indicates the 95% confidence interval

Figure 4 exhibited the relationships between NIRA and the threshold and EI centrality of each symptom. In Fig. 4A, the NIRA outcomes in alleviating interventions were associated with thresholds (*r* = 0.54, *p* < 0.05) rather than EI values (*r* = 0.07, *p* = 0.81). Symptoms with higher thresholds (more likely to be absent) were more likely to be targeted for alleviating interventions. In Fig. 4B, the NIRA outcomes for aggravating interventions were largely related to thresholds (*r* = −0.95, *p* < 0.001) and EI values (*r* = 0.61, *p* < 0.05). Symptoms with lower thresholds (more likely to be absent) or higher EI values (more influential in the network) were more likely to be targeted for aggravating interventions.

## Discussion

This study used network analysis to explore the comorbidity of anxiety and depression among Chinese healthcare workers one year after the end of the dynamic zero-COVID policy and identified effective targets for intervention. In our anxiety-depression Ising network, we identified central symptoms such as “guilt” and “appetite changes”, as well as bridge symptoms like “guilt” and “excessive worry”. Simulation interventions pointed to “Anhedonia” as a key target for treatment and “guilt” as a key target for prevention. These findings shed light on the ongoing psychological challenges healthcare workers faced post-zero-COVID and offer valuable insights for targeted interventions.

### Central symptoms in the anxiety-depression network: guilt and appetite changes

“Guilt,” affecting around 20% of healthcare workers, emerged as a central symptom in the anxiety-depression network. This differs from previous studies during the dynamic zero-COVID period [10, 11], but guilt remains a central symptom in anxiety-depression network in various groups, including adolescents [31] and young adults [32]. According to the Diagnostic and Statistical Manual of Mental Disorders, Fifth Edition (DSM-5), guilt is one of the common symptoms of depression, typically manifesting as excessive self-blame or feelings of failure, worthlessness, and the belief that one has let oneself or others down [33]. As the unique characterizes of their profession, healthcare workers often experienced guilty for various complex reasons. During the pandemic, they felt guilt related to family, work, and the risk of infection during the pandemic [34]. Family- and work-related guilt persisted even after the crisis, as long working hours and heavy workloads created work-to-family conflict, making them feel guilty for not meeting family needs [35]. Additionally, empathy for patients led to pathogenic guilt, with healthcare workers feeling responsible for patients’ suffering, even when they were powerless to help [36, 37]. This prolonged guilt worsened when patients’ conditions deteriorated [38], when deaths occurred [39], or after medical errors happened [40]. Along with guilt, shame, anger, disgust, anxiety, loss of confidence, and sadness were also common emotions related to moral injury in healthcare [41]. Consequently, healthcare workers had to engage in extra emotional labor and prioritize the needs and emotions of patients over their own [42, 43]. Over time, this prolonged guilt led to physical and emotional exhaustion [36], worsening other depression and anxiety symptoms, and even contributing to suicidal thoughts [44, 45]. These dynamics explain the associations between guilt and symptoms like sleep difficulties, fatigue, motor disturbances, and suicidal ideation in our network. Our findings also align with Zou et al., who found the mutually reinforcing relationships between guilt and depressive symptoms [46].

“Appetite changes,” recognized as a common depressive symptom [33], also emerged as a central symptom in the network. Unlike previous studies that highlighted pandemic-related anxiety [10, 11], our finding indicated that disrupted eating habits might be a core symptom leading to depression and anxiety among healthcare workers in the post-dynamic zero-COVID policy era. About 40% of healthcare workers in our sample reported abnormal increases or decreases in appetite, which might be explained by the high levels of work pressures during this period [47, 48]. Irregular work schedules often disrupted normal eating patterns and sleep routines, leading to hormonal imbalances and altered energy metabolism [49–51]. Our research found significant connections between appetite changes, anhedonia, depressed or sad mood, and motor disturbances, which supported earlier findings. Studies have regarded appetite changes as a direct manifestation of anhedonia [52–54]. Keränen et al. found that anhedonia was associated with uncontrolled eating, emotional eating, and binge eating [55]. Additionally, Mason et al. proposed a model of binge-eating disorder (BED), explaining how anhedonia, low mood, and lack of motivation interact to maintain binge-eating behavior [56].

### Bridge symptoms of the anxiety-depression network: guilt and excessive worry

“Guilt” within depressive symptoms and “Excessive worry” within anxiety symptoms emerged as two bridge symptoms in the network, linking clusters of depression and anxiety symptoms. In our sample, approximately 35% of healthcare workers reported experiencing “excessive worry” about everyday events or activities. These newly identified bridge symptoms differ from those reported in previous studies conducted during the dynamic zero-COVID period [10, 13, 57]. However, our results aligned with Chen et al. [14], who found guilt to be a key bridge symptom between depression and anxiety among Chinese mental health professionals after the zero-COVID period. These changes reflect a new pattern of comorbid anxiety and depression as the pandemic eased and work returned to normal. During this period, healthcare workers commonly felt guilt and worry due to family-work conflict rather than infection. In their daily work, healthcare workers often felt guilt and anger for not providing optimal care, worried about treatment failures, and felt frustrated by unmet outcomes [58]. Their empathy-based guilt towards patients made them especially concerned about future medical errors [45]. When errors occurred, they might suffer severe emotional distress, such as guilt, insomnia, anxiety, and concentration difficulties, and some even risked job loss or contemplated suicide [44, 59]. Contradictorily, even when returning home after work, these feelings seemed difficult to improve and might even continue to worsen [60]. Regardless of the pandemic, healthcare workers often faced family conflicts and impaired family functioning [17], which further increased their feelings of guilt, worry, or shame for not being present in their family roles [35, 60]. Moreover, guilt and excessive worry were highly related to rumination [61, 62], which is a critical factor contributing to the comorbidity of anxiety and depression [63].

More concerningly, stigma, fear of negative career consequences, and lack of time commonly hinder healthcare workers from seeking mental health support [64, 65], potentially exacerbating feelings of guilt and worry. Despite their medical expertise, many healthcare workers remain affected by mental illness stigma and view mental health diagnoses and treatment as embarrassing and shameful [66, 67]. Perceived stigma often led to their self-stigma, leading to self-blame for perceived failures as caregivers and concerns about damage to their professional image [68, 69]. Healthcare workers also worry that disclosing mental health could harm their job applications, medical license, and career advancement [64, 70, 71]. Additionally, taking time off to prioritize mental health may evoke guilt toward colleagues and patients due to staff shortages and a strong sense of duty [65, 69]. Limited time also reduce their willingness to participate in therapies such as counseling or cognitive behavioral therapy, which typically require multiple sessions to be effective [67]. In sum, these barriers undermine help-seeking behaviors and may accelerate the progression of comorbid depression and anxiety.

### Target symptoms for the treatment and prevention of depressive and anxiety symptoms

Using the NIRA, this study identified effective targets for both alleviating and aggravating interventions in the anxiety-depression network. First, “Anhedonia” was the most effective target for treatment, as alleviating it decreased the overall severity of the network to an extreme. This could be explained by the threshold of “Anhedonia” (natural disposition for manifestation) rather than EI (influence on other symptoms). “Anhedonia” had a negative threshold close to 0 (albeit not the closest), suggesting it tended to remain absent but was near activated. Anhedonia affected nearly 70% of healthcare workers and was the most prevalent symptom in our sample. Although not the core or bridge symptom in our network, anhedonia has been found to promote the anxiety-depression comorbidity in adults [72]. Healthcare workers often face work pressure, promotion and evaluation challenges, and difficulty balancing family and career, which weaken their response to rewards and led to anhedonia. Therefore, it is necessary for hospitals to provide systematic support for healthcare workers, including optimizing working conditions, reducing workloads, and strengthening reward mechanisms [73, 74]. Sufficient time outside of work can help them develop personalized lifestyles to alleviate anhedonia. Social activities, outdoor physical exercise, and spending time on television, the internet, or social media, have been found effective in improving pleasure and positive affects among anhedonic adults [75, 76]. Moreover, hospitals should organize psychological intervention programs to help manage anhedonia, such as behavioral activation, mindfulness, and gratitude writing [77, 78].

In addition to being a central and bridge symptom, “guilt” was the most effective target for prevention, as aggravating it furthest increased the overall severity of the network. This result fitted the target for prevention, which is often something that hasn’t happened yet but could led serious consequence. Due to its lowest threshold and prevalence among all symptoms, guilt is the least likely to be activated, acting as a “last safe zone”. However, it plays a key role in linking symptoms and driving anxiety-depression comorbidity. If guilt worsens, it can cause the entire network to deteriorate. To prevent this, healthcare workers should engage in surface-level emotional labor to prevent burnout, such as showing concern for patients without excessive empathy [79]. Hospitals should reduce their workloads to ensure they have time to care for both their families and their own mental well-being [35, 65]. Furthermore, organizing lectures or science outreach programs concerning mental health can help normalize the need for psychological support and reduce stigma among healthcare workers [65]. Additionally, contemplative practices, such as mindfulness, Tibetan, and Theravada, may also help prevent pathological guilt [80].

### Implications

This study examined the mental health of Chinese healthcare workers in the post-zero-COVID era, revealing new mechanisms of anxiety and depression comorbidity. Our findings highlighted the role of guilt and excessive worry in reinforcing comorbidity, as well as the central roles of guilt and appetite changes. As the impact of the pandemic decreased, the focus of these symptoms shifted to family and work-related issues. Our study also highlights the psychological effects of pandemic-related policy changes on healthcare professionals and emphasizes the need for timely, context-specific psychological interventions. By using the NIRA algorithm based on the Ising network, we identified practical intervention strategies. Depression and anxiety symptoms in healthcare workers are often dismissed as mere occupational fatigue, making effective intervention difficult. However, our network analysis approach identifies specific targets for intervention, such as guilty and anhedonia. Experimental evidence supports that behavioral activation therapy (BATA) and mindfulness-based cognitive therapy (MBCT) can effectively reduce anhedonia and guilt [81, 82]. In addition to individual interventions, establishing comprehensive support systems—including emotional regulation programs, early screening mechanisms, and a supportive work environment—is crucial for enhancing healthcare workers'resilience and well-being.

### Limitations

Some limitations of this study should be noted. First, the cross-sectional Ising network reveals the correlations between symptoms rather than causal relationships. Future research should employ longitudinal designs to model cross-lagged panel network (CLPN) of anxiety and depression symptoms among healthcare workers, in order to determine whether these relationships are unidirectional or bidirectional [83]. Furthermore, future studies could incorporate simulated interventions targeting identified symptoms and assess the alleviation effects over time. Specifically, researchers might aim to reduce the severity of the target symptom (e.g., anhedonia), or weaken the associations between that symptom and others (e.g., anhedonia and appetite changes) to disrupt the overall symptom network [84]. Second, the findings are based on a single-region sample, which may be influenced by local geographic, economic, and cultural factors, thus limiting the generalizability. However, healthcare workers, regardless of region or culture, were among the most affected groups during the pandemic, highlighting the importance of addressing their mental health [85]. Future studies should examine healthcare workers’ mental health across diverse regions and cultures to provide more comprehensive insights and inform the development of targeted interventions. Third, the current sample included clinical doctors and nurses as well as non-clinical staff (e.g., technicians and administrators). However, we did not collect detailed information on occupational categories, and therefore cannot compare mental health outcomes across different roles. This limitation highlights the need for caution when generalizing the findings to specific clinical groups. Future research should examine how different healthcare roles experience psychological distress to inform more targeted interventions.

## Conclusion

Although the COVID-19 situation in China has eased after the end of the dynamic zero-COVID policy, healthcare workers still face immense pressure from the aftermath of the pandemic, new threats, and emerging health challenges. Using network analysis, this study explored the comorbidity of anxiety and depression among healthcare workers in this new period and further identified effective targets for intervention. In the anxiety-depression Ising network, we identified central symptoms such as “guilt” and “appetite changes”, as well as bridge symptoms like “guilt” and “excessive worry”. Using NIRA, simulated interventions pointed to “Anhedonia” as a key target for treatment and “guilt” as a key target for prevention. These findings shed light on the ongoing psychological challenges healthcare workers faced post-zero-COVID and offer valuable insights for targeted interventions.

## Supplementary Information

Supplementary Material 1.

## Data Availability

The data for this work will be available upon request. The analytic code can be found in OSF (https://osf.io/nsd38/?view_only = 5ce1808a02c74 d0fa72 d9af30916aae5).

## Abbreviations

PHQ-9: Patient Health Questionnaire-9
GAD-7: Generalized Anxiety Disorder-7
NIRA: *NodeIdentifyR* Algorithm
EI: Expected influence
COVID: Coronavirus
CS-C: Correlation stability coefficient
DSM-5: Diagnostic and Statistical Manual of Mental Disorders, Fifth Edition
BATA: Behavioral activation therapy
MBCT: Mindfulness-based cognitive therapy

## Footnotes

1 The additional partial correlation network based on continuous scores for each symptom was shown in **Figure S1**). Mantel’s statistic further suggested a high similarity between the two network matrices (*r* =.78, *p* =.001). Thus, the Ising network accurately reflects the relationships between symptoms similar to partial correlation network and did not impact the network’s sensitivity.

## References

[1] Zhou R-f. Sun K, Fang X, LU H-z: Development of COVID-19 pandemic prevention and control policies in China. Fudan Univ J Med Sci. 2024;51(1):109–14.

[2] Song Y-Y, Ling X, Dan L, Mei F, Cui Y, Yan J, and Wu Y. Depression and Anxiety Symptoms and Their Associated Factors Among Chinese Residents After the Lifting of the Dynamic Zero-COVID Policy: A Cross-Sectional Study. Int J Gen Med. 2023;16(null):5921–5934. 10.2147/IJGM.S442093 PMC10725747 38106974

[3] Jiao T, Huang Y, Sun H, Yang L. Research progress of post-acute sequelae after SARS-CoV-2 infection. Cell Death Dis. 2024;15(4):257. 38605011 10.1038/s41419-024-06642-5 PMC11009241

[4] Xue J, Xu D, Shan X. Research progress on common long-term sequelae of CovID-19 rehabilitation patients and corresponding measures for health management. South Chin J Prevent Med. 2024;50(2):191–194,198.

[5] 全国新型冠状病毒感染疫情情况 (2023年11月) (National Situation of Novel Coronavirus Infection, November 2023) [https://www.chinacdc.cn/jksj/xgbdyq/202410/t20241025_302090.html]

[6] Yue T-y, Kang L-h. Psychological status of first-line health care workers after COVID-19 epidemic at the end of 2022. Chin J Nosocomiol. 2024;34(16):2531–5.

[7] Lunansky G, Naberman J, van Borkulo CD, Chen C, Wang L, Borsboom D. Intervening on psychopathology networks: Evaluating intervention targets through simulations. Methods. 2022;204:29–37. 34793976 10.1016/j.ymeth.2021.11.006

[8] Borsboom D, Cramer AOJ. Network Analysis: An Integrative Approach to the Structure of Psychopathology. Annu Rev Clin Psychol. 2013;9:91–121. 23537483 10.1146/annurev-clinpsy-050212-185608

[9] Johns G, Samuel V, Freemantle L, Lewis J, Waddington L. The global prevalence of depression and anxiety among doctors during the covid-19 pandemic: Systematic review and meta-analysis. J Affect Disord. 2022;298:431–41. 34785264 10.1016/j.jad.2021.11.026 PMC8596335

[10] Jin Y, Sha S, Tian T, Wang Q, Liang S, Wang Z, Liu Y, Cheung T, Su Z, Ng CH, et al. Network analysis of comorbid depression and anxiety and their associations with quality of life among clinicians in public hospitals during the late stage of the COVID-19 pandemic in China. J Affect Disord. 2022;314:193–200. 35780965 10.1016/j.jad.2022.06.051 PMC9242942

[11] Peng P, Chen Q, Liang M, Liu Y, Chen S, Wang Y, Yang Q, Wang X, Li M, Wang Y, et al. A network analysis of anxiety and depression symptoms among Chinese nurses in the late stage of the COVID-19 pandemic. Front Public Health. 2022;10:996386. 36408014 10.3389/fpubh.2022.996386 PMC9667894

[12] Hummel S, Oetjen N, Du J, Posenato E, Resende de Almeida RM, Losada R, Ribeiro O, Frisardi V, Hopper L, Rashid A et al. Mental Health Among Medical Professionals During the COVID-19 Pandemic in Eight European Countries: Cross-sectional Survey Study. J Med Internet Res. 2021;23(1):e24983. 10.2196/24983 PMC7817254 33411670

[13] Ren L, Wang Y, Wu L, Wei Z, Cui L-B, Wei X, Hu X, Peng J, Jin Y, Li F, et al. Network structure of depression and anxiety symptoms in Chinese female nursing students. BMC Psychiatry. 2021;21(1):279. 34059013 10.1186/s12888-021-03276-1 PMC8168020

[14] Chen M-Y, Chen P, An F-R, Sha S, Feng Y, Su Z, Cheung T, Ungvari GS, Ng CH, Zhang L, et al. Depression, anxiety and suicidality among Chinese mental health professionals immediately after China’s dynamic zero-COVID policy: A network perspective. J Affect Disord. 2024;352:153–62. 38316260 10.1016/j.jad.2024.01.270

[15] Zeng N, Zhao Y-M, Yan W, Li C, Lu Q-D, Liu L, Ni S-Y, Mei H, Yuan K, Shi L, et al. A systematic review and meta-analysis of long term physical and mental sequelae of COVID-19 pandemic: call for research priority and action. Mol Psychiatry. 2023;28(1):423–33. 35668159 10.1038/s41380-022-01614-7 PMC9168643

[16] 2023年我国卫生健康事业发展统计公报 (China's Health Industry Development Statistical Bulletin for 2023) [https://www.gov.cn/lianbo/bumen/202408/content_6971241.htm]

[17] Tekin S, Nicholls H, Lamb D, Glover N, Billings J. Impact of occupational stress on healthcare workers’ family members before and during COVID-19: A systematic review. PLoS ONE. 2024;19(9):e0308089. 39298458 10.1371/journal.pone.0308089 PMC11412678

[18] Li P, Huang N, Yang X, Fang Y, Chen Z. A simulation-based network analysis of intervention targets for adolescent depressive and anxiety symptoms. Asian J Psychiatr. 2024;99:104152. 39018702 10.1016/j.ajp.2024.104152

[19] Wang S, Hou W, Tao Y, Ma Z, Li K, Wang Y, Xu Z, Liu X, Zhang L. Mapping network connection among symptoms of anxiety, depression, and sleep disturbance in Chinese high school students. Front Public Health. 2022;10:1015166. 36466464 10.3389/fpubh.2022.1015166 PMC9710521

[20] Zhang L, Tao Y, Hou W, Niu H, Ma Z, Zheng Z, Wang S, Zhang S, Lv Y, Li Q, et al. Seeking bridge symptoms of anxiety, depression, and sleep disturbance among the elderly during the lockdown of the COVID-19 pandemic-A network approach. Front Psych. 2022;13:919251. 10.3389/fpsyt.2022.919251 PMC9381922 35990065

[21] Kroenke K, Spitzer RL, Williams JB. The PHQ-9: validity of a brief depression severity measure. J Gen Intern Med. 2001;16(9):606–13. 11556941 10.1046/j.1525-1497.2001.016009606.x PMC1495268

[22] Spitzer RL, Kroenke K, Williams JBW, Löwe B. A Brief Measure for Assessing Generalized Anxiety Disorder: The GAD-7. Arch Intern Med. 2006;166(10):1092–7. 16717171 10.1001/archinte.166.10.1092

[23] R Core Team. R: A Language and Environment for Statistical Computing. In: R Foundation for Statistical Computing. Vienna; 2023.

[24] Jones PJ, Ma R, McNally RJ. Bridge Centrality: A Network Approach to Understanding Comorbidity. Multivar Behav Res. 2021;56(2):353–67. 10.1080/00273171.2019.1614898 31179765

[25] Epskamp S, Cramer AO, Waldorp LJ, Schmittmann VD, Borsboom D. qgraph: Network visualizations of relationships in psychometric data. J Stat Softw. 2012;48:1–18.

[26] Fortin M-J, Gurevitch J. Mantel tests: spatial structure in field experiments. Design and analysis of ecological experiments. 1993. p. 342–59.

[27] Opsahl T, Agneessens F, Skvoretz J. Node centrality in weighted networks: Generalizing degree and shortest paths. Social networks. 2010;32(3):245–51.

[28] Tang Q, He X, Zhang L, Liu X, Tao Y, Liu G. Effects of Neuroticism on Differences in Symptom Structure of Life Satisfaction and Depression-Anxiety among College Students: A Network Analysis. Behav Sci. 2023;13(8):641. 37622781 10.3390/bs13080641 PMC10451887

[29] Epskamp S, Borsboom D, Fried EI. Estimating psychological networks and their accuracy: A tutorial paper. Behav Res Methods. 2018;50(1):195–212. 28342071 10.3758/s13428-017-0862-1 PMC5809547

[30] Bringmann LF, Elmer T, Epskamp S, Krause RW, Schoch D, Wichers M, Wigman JT, Snippe E. What do centrality measures measure in psychological networks? J Abnorm Psychol. 2019;128(8):892–903. 31318245 10.1037/abn0000446

[31] Cai H, Bai W, Liu H, Chen X, Qi H, Liu R, Cheung T, Su Z, Lin J, Tang Y-l et al. Network analysis of depressive and anxiety symptoms in adolescents during the later stage of the COVID-19 pandemic. Transl Psychiatry. 2022;12(1):98. 10.1038/s41398-022-01838-9 PMC8907388 35273161

[32] Zhao Y, Qu D, Chen S, Chi X. Network analysis of internet addiction and depression among Chinese college students during the COVID-19 pandemic: A longitudinal study. Comput Human Behav. 2023;138:107424. 35945974 10.1016/j.chb.2022.107424 PMC9352366

[33] American Psychiatric Association. Diagnostic and statistical manual of mental disorders: DSM-5, vol. 5. Washington, DC: American psychiatric association; 2013.

[34] Fischer IC, Norman SB, Feder A, Feingold JH, Peccoralo L, Ripp J, Pietrzak RH. Downstream consequences of moral distress in COVID-19 frontline healthcare workers: Longitudinal associations with moral injury-related guilt. Gen Hosp Psychiatry. 2022;79:158–61. 36403350 10.1016/j.genhosppsych.2022.11.003 PMC9664834

[35] Chen S, Cheng M-I. Exploring the reciprocal nature of work-family guilt and its effects on work/family-related performance. Community Work Family. 2023. p. 1–16.

[36] Duarte J, Pinto-Gouveia J. Empathy and feelings of guilt experienced by nurses: A cross-sectional study of their role in burnout and compassion fatigue symptoms. Appl Nurs Res. 2017;35:42–7. 28532725 10.1016/j.apnr.2017.02.006

[37] O’ Connor LE, Berry JW, Lewis TB, Stiver DJ. Empathy-based pathogenic guilt, pathological altruism, and psychopathology. In: Oakley B, Knafo A, Madhavan G, Wilson DS, editors. Pathological altruism. New York: Oxford University Press; 2012. p. 10–30.

[38] Walsh M. The experience of witnessing patients' trauma and suffering for acute care nurses. Master's thesis. University of British Columbia; 2009.

[39] Tu J, Shen M, Li Z. When cultural values meets professional values: a qualitative study of chinese nurses’ attitudes and experiences concerning death. BMC Palliat Care. 2022;21(1):181. 36242029 10.1186/s12904-022-01067-3 PMC9561326

[40] Delbanco T, Bell SK. Guilty, Afraid, and Alone — Struggling with Medical Error. N Engl J Med. 2007;357(17):1682–3. 17960011 10.1056/NEJMp078104

[41] Rabin S, Kika N, Lamb D, Murphy D, Am Stevelink S, Williamson V, Wessely S, Greenberg N. Moral Injuries in Healthcare Workers: What Causes Them and What to Do About Them? J Healthcare Leadership. 2023;15:153–60. 10.2147/JHL.S396659 PMC10440078 37605753

[42] Kirk K, Cohen L, Edgley A, Timmons S. “I don’t have any emotions”: An ethnography of emotional labour and feeling rules in the emergency department. J Adv Nurs. 2021;77(4):1956–67. 33576110 10.1111/jan.14765

[43] Funk LM, Peters S, Roger KS. The Emotional Labor of Personal Grief in Palliative Care: Balancing Caring and Professional Identities. Qual Health Res. 2017;27(14):2211–21. 28891373 10.1177/1049732317729139

[44] Robertson JJ, Long B. Suffering in Silence: Medical Error and its Impact on Health Care Providers. J Emerg Med. 2018;54(4):402–9. 29366616 10.1016/j.jemermed.2017.12.001

[45] Waterman AD, Garbutt J, Hazel E, Dunagan WC, Levinson W, Fraser VJ, Gallagher TH. The Emotional Impact of Medical Errors on Practicing Physicians in the United States and Canada. Joint Commission J Qual Patient Safety. 2007;33(8):467–76. 10.1016/s1553-7250(07)33050-x 17724943

[46] Zou H, Gao J, Wu W, Huo L, Zhang W. Which comes first? Comorbidity of depression and anxiety symptoms: A cross-lagged network analysis. Soc Sci Med. 2024;360:117339. 39393294 10.1016/j.socscimed.2024.117339

[47] Yaman GB, Hocaoğlu Ç. Examination of eating and nutritional habits in health care workers during the COVID-19 pandemic. Nutrition. 2023;105:111839. 36270134 10.1016/j.nut.2022.111839 PMC9439855

[48] Wolska A, Stasiewicz B, Kaźmierczak-Siedlecka K, Ziętek M, Solek-Pastuszka J, Drozd A, Palma J, Stachowska E. Unhealthy food choices among healthcare shift workers: a cross-sectional study. Nutrients. 2022;14(20):4327. 36297011 10.3390/nu14204327 PMC9611829

[49] Lok R, Qian J, Chellappa SL. Sex differences in sleep, circadian rhythms, and metabolism: Implications for precision medicine. Sleep Med Rev. 2024;75:101926. 38564856 10.1016/j.smrv.2024.101926

[50] Rogers EM, Banks NF, Jenkins NDM. The effects of sleep disruption on metabolism, hunger, and satiety, and the influence of psychosocial stress and exercise: A narrative review. Diabetes Metab Res Rev. 2024;40(2):e3667. 37269143 10.1002/dmrr.3667

[51] St-Onge M-P. Sleep–obesity relation: underlying mechanisms and consequences for treatment. Obes Rev. 2017;18(S1):34–9. 28164452 10.1111/obr.12499 PMC13098705

[52] Simmons WK, Burrows K, Avery JA, Kerr KL, Bodurka J, Savage CR, Drevets WC. Depression-Related Increases and Decreases in Appetite: Dissociable Patterns of Aberrant Activity in Reward and Interoceptive Neurocircuitry. Am J Psychiatry. 2016;173(4):418–28. 26806872 10.1176/appi.ajp.2015.15020162 PMC4818200

[53] Hyldelund NB, Byrne DV, Chan RCK, Andersen BV. The Relationship between Social Anhedonia and Perceived Pleasure from Food—An Exploratory Investigation on a Consumer Segment with Depression and Anxiety. Foods. 2022;11(22):3659. 36429251 10.3390/foods11223659 PMC9689578

[54] Cho J, Goldenson NI, Pester MS, Khoddam R, Bello MS, Dunton GF, Belcher BR, Leventhal AM. Longitudinal Associations Between Anhedonia and Body Mass Index Trajectory Groups Among Adolescents. J Adolesc Health. 2018;63(1):81–7. 29731318 10.1016/j.jadohealth.2017.12.022 PMC6067955

[55] Keränen A-M, Rasinaho E, Hakko H, Savolainen M, Lindeman S. Eating behavior in obese and overweight persons with and without anhedonia. Appetite. 2010;55(3):726–9. 20801180 10.1016/j.appet.2010.08.012

[56] Mason TB, Smith KE, Anderson LM, Hazzard VM. Anhedonia, positive affect dysregulation, and risk and maintenance of binge-eating disorder. Int J Eat Disord. 2021;54(3):287–92. 33295671 10.1002/eat.23433 PMC8673784

[57] Bai W, Xi H-T, Zhu Q, Ji M, Zhang H, Yang B-X, Cai H, Liu R, Zhao Y-J, Chen L, et al. Network analysis of anxiety and depressive symptoms among nursing students during the COVID-19 pandemic. J Affect Disord. 2021;294:753–60. 34375200 10.1016/j.jad.2021.07.072 PMC8433813

[58] Kolehmainen N, McAnuff J. “I should have discharged him but I felt guilty”: a qualitative investigation of clinicians’ emotions in the context of implementing occupational therapy. Implement Sci. 2014;9(1):141. 25273675 10.1186/s13012-014-0141-9 PMC4198626

[59] Gallagher TH, Waterman AD, Ebers AG, Fraser VJ, Levinson W. Patients’ and Physicians’ Attitudes Regarding the Disclosure of Medical Errors. JAMA. 2003;289(8):1001–7. 12597752 10.1001/jama.289.8.1001

[60] Pan Y, Yang Xh, He JP, Gu YH, Zhan XL, Gu HF, Qiao QY, Zhou DC, Jin HM. To be or not to be a doctor, that is the question: a review of serious incidents of violence against doctors in China from 2003–2013. J Public Health. 2015;23(2):111–6.

[61] Everaert J, Joormann J. Emotion Regulation Difficulties Related to Depression and Anxiety: A Network Approach to Model Relations Among Symptoms, Positive Reappraisal, and Repetitive Negative Thinking. Clin Psychol Sci. 2019;7(6):1304–18.

[62] Leonardi J, Fimiani R, Faccini F, Gorman BS, Bush M, Gazzillo F. An Empirical Investigation into Pathological Worry and Rumination: Guilt, Shame, Depression, and Anxiety. Psychology Hub. 2020;37(3):31–42.

[63] Spinhoven P, van Hemert AM, Penninx BW. Repetitive negative thinking as a mediator in prospective cross-disorder associations between anxiety and depression disorders and their symptoms. J Behav Ther Exp Psychiatry. 2019;63:6–11. 30551055 10.1016/j.jbtep.2018.11.007

[64] Zaman N, Mujahid K, Ahmed F, Mahmud S, Naeem H, Riaz U, Ullah U, Cox B. What are the barriers and facilitators to seeking help for mental health in NHS doctors: a systematic review and qualitative study. BMC Psychiatry. 2022;22(1):595. 36071392 10.1186/s12888-022-04202-9 PMC9450826

[65] Maple J-L, Willis K, Lewis S, Putland M, Baldwin P, Bismark M, Harrex W, Johnson D, Karimi L, Smallwood N. Healthcare workers’ perceptions of strategies supportive of their mental health. J Med Surg Public Health. 2024;2:100049.

[66] Clement S, Schauman O, Graham T, Maggioni F, Evans-Lacko S, Bezborodovs N, Morgan C, Rüsch N, Brown JSL, Thornicroft G. What is the impact of mental health-related stigma on help-seeking? A systematic review of quantitative and qualitative studies. Psychol Med. 2015;45(1):11–27. 24569086 10.1017/S0033291714000129

[67] Gold KJ, Andrew LB, Goldman EB, Schwenk TL. “I would never want to have a mental health diagnosis on my record”: A survey of female physicians on mental health diagnosis, treatment, and reporting. Gen Hosp Psychiatry. 2016;43:51–7. 27796258 10.1016/j.genhosppsych.2016.09.004

[68] Jones N, Whybrow D, Coetzee R. UK military doctors; stigma, mental health and help-seeking: a comparative cohort study. J R Army Med Corps. 2018;164(4):259. 29523754 10.1136/jramc-2018-000928

[69] Spiers J, Buszewicz M, Chew-Graham CA, Gerada C, Kessler D, Leggett N, Manning C, Taylor AK, Thornton G, Riley R. Barriers, facilitators, and survival strategies for GPs seeking treatment for distress: a qualitative study. Br J Gen Pract. 2017;67(663):e700–8. 28893766 10.3399/bjgp17X692573 PMC5604834

[70] Mehta SS, Edwards ML. Suffering in Silence: Mental Health Stigma and Physicians’ Licensing Fears. Am J Psychiatry Residents J. 2018;13(11):2–4.

[71] Taylor CJ, Wright M, Jackson CL, Hobbs R. Grass is greener? General practice in England and Australia. Br J Gen Pract. 2016;66(649):428. 27481974 10.3399/bjgp16X686377 PMC4979942

[72] Winer ES, Bryant J, Bartoszek G, Rojas E, Nadorff MR, Kilgore J. Mapping the relationship between anxiety, anhedonia, and depression. J Affect Disord. 2017;221:289–96. 28668590 10.1016/j.jad.2017.06.006 PMC6080718

[73] Zhang J, Wang Y, Xu J, You H, Li Y, Liang Y, Li S, Ma L, Lau JT-f, Hao Y *et al*: Prevalence of mental health problems and associated factors among front-line public health workers during the COVID-19 pandemic in China: an effort–reward imbalance model-informed study. BMC Psychol. 2021;9(1):55. 10.1186/s40359-021-00563-0 PMC8040352 33845895

[74] Olson K, Marchalik D, Farley H, Dean SM, Lawrence EC, Hamidi MS, Rowe S, McCool JM, O’Donovan CA, Micek MA, et al. Organizational strategies to reduce physician burnout and improve professional fulfillment. Curr Probl Pediatr Adolesc Health Care. 2019;49(12):100664. 31588019 10.1016/j.cppeds.2019.100664

[75] Van Roekel E, Vrijen C, Heininga VE, Masselink M, Bos EH, Oldehinkel AJ. An Exploratory Randomized Controlled Trial of Personalized Lifestyle Advice and Tandem Skydives as a Means to Reduce Anhedonia. Behav Ther. 2017;48(1):76–96. 28077223 10.1016/j.beth.2016.09.009

[76] Sun C-w, Wang Y-j, Fang Y-q, He Y-q. Wang X, So BCL, Shum DHK, Yan C: The effect of physical activity on anhedonia in individuals with depressive symptoms. PsyCh Journal. 2022;11(2):214–26. 34530499 10.1002/pchj.485

[77] Potsch L, Rief W. Effectiveness of behavioral activation and mindfulness in increasing reward sensitivity and reducing depressive symptoms - A randomized controlled trial. Behav Res Ther. 2024;173:104455. 38128402 10.1016/j.brat.2023.104455

[78] Limpächer C, Kindt T, Hoyer J. Counteract Anhedonia! Introducing an Online-Training to Enhance Reward Experiencing – A Pilot Study. Clinl Psychol Eur. 2024;6(2):1–20. 10.32872/cpe.13751 PMC11303914 39119054

[79] Pandey J, Singh M. Donning the mask: effects of emotional labour strategies on burnout and job satisfaction in community healthcare. Health Policy Plan. 2016;31(5):551–62. 26491059 10.1093/heapol/czv102

[80] O’Connor LE, Rangan RK, Berry JW, Stiver DJ, Rick H, Ark W, Li T. Empathy, compassionate altruism and psychological well-being in contemplative practitioners across five traditions. Psychology. 2015;6(8):989–1000.

[81] Scutari M, Nagarajan R. Identifying significant edges in graphical models of molecular networks. Artif Intell Med. 2013;57(3):207–17. 23395009 10.1016/j.artmed.2012.12.006 PMC4070079

[82] Viki GT, Osgood D, Phillips S. Dehumanization and self-reported proclivity to torture prisoners of war. J Exp Soc Psychol. 2013;49(3):325–8.

[83] Rhemtulla M, van Bork R, Cramer A. Cross-lagged network models. Multivariate Behav Res. 2022.

[84] Borsboom D, Deserno MK, Rhemtulla M, Epskamp S, Fried EI, McNally RJ, Robinaugh DJ, Perugini M, Dalege J, Costantini G, et al. Network analysis of multivariate data in psychological science. Nat Rev Methods Primers. 2021;1(1):58.

[85] Dong Y, Yeo MC, Tham XC, Danuaji R, Nguyen TH, Sharma AK, Rn K, Pv M, Tai M-LS, Ahmad A et al. Investigating Psychological Differences Between Nurses and Other Health Care Workers From the Asia-Pacific Region During the Early Phase of COVID-19: Machine Learning Approach. JMIR Nurs. 2022;5(1):e32647. 10.2196/32647 PMC9162133 35648464

