# Supplementary material for: A simulation-based network analysis of intervention targets for comorbid symptoms of depression and anxiety in Chinese healthcare workers in the post-dynamic zero-COVID policy era
Source: BMC Psychiatry. 2025 May 6;25:457. doi: 10.1186/s12888-025-06931-z (PMC12057238; doi:10.1186/s12888-025-06931-z)
Supplement: Supplementary file 1 — Supplementary Material 1. [file 12888_2025_6931_MOESM1_ESM.docx]

**Online Supplemental Materials**

**Tables**

**Table S1. Descriptive statistics of variables and Group Comparisons.**

| **Variable** | ***N*** | | **Anxiety (GAD-7)** | | | |  | **Depression (PHQ-9)** | | | |
| --- | --- | --- | --- | --- | --- | --- | --- | --- | --- | --- | --- |
|  |  |  | ***M*** | ***SD*** | ***F*/*t*** | ***p*** |  | ***M*** | ***SD*** | ***F*/*t*** | ***p*** |
| **Gender** |  |  |  |  | 4.59 | **< .001** |  |  |  | 3.59 | **< .001** |
| Male | 204 |  | 4.75 | 4.46 |  |  |  | 4.94 | 5.49 |  |  |
| Female | 504 |  | 3.43 | 2.99 |  |  |  | 3.69 | 3.52 |  |  |
| **Marriage** |  |  |  |  | 18.01 | **< .001** |  |  |  | 12.54 | **< .001** |
| Married | 543 |  | 3.79 | 3.43 |  |  |  | 3.98 | 4.08 |  |  |
| Never-married | 133 |  | 3.09 | 2.91 |  |  |  | 3.49 | 3.26 |  |  |
| Divorced, widowed, or others | 32 |  | 7.16 | 5.29 |  |  |  | 7.53 | 7.44 |  |  |
| **Income per year (in RMB)** |  |  |  |  | 20.06 | **< .001** |  |  |  | 10.48 | **< .001** |
| Under 18,000 | 164 |  | 2.59 | 1.88 |  |  |  | 2.63 | 2.32 |  |  |
| 18,000-60,000 | 379 |  | 3.83 | 3.49 |  |  |  | 4.18 | 4.33 |  |  |
| 60,000-96,000 | 148 |  | 4.57 | 4.22 |  |  |  | 5.17 | 4.86 |  |  |
| Above 96,000 | 17 |  | 8.41 | 4.39 |  |  |  | 5.06 | 6.21 |  |  |

***Note.*** *M*, mean; *SD*, standard deviation.

**Table S2: The edge-weight matrix and the Threshold indices of the Anxiety-Depression Symptom Network.**

|  | **Edge-weight matrix** | | | | | | | | | | | | | | | |  | **Threshold** |
| --- | --- | --- | --- | --- | --- | --- | --- | --- | --- | --- | --- | --- | --- | --- | --- | --- | --- | --- |
|  | GAD1 | GAD2 | GAD3 | GAD4 | GAD5 | GAD6 | GAD7 | PHQ1 | PHQ2 | PHQ3 | PHQ4 | PHQ5 | PHQ6 | PHQ7 | PHQ8 | PHQ9 |  |  |
| **Anxiety** |  |  |  |  |  |  |  |  |  |  |  |  |  |  |  |  |  |  |
| GAD1 | 0 | 0.477 | 0 | 0 | -0.362 | 0.720 | 2.103 | 0 | 0 | 0 | 0.294 | 0 | 0 | 0 | 0 | 0.308 |  | -1.24 |
| GAD2 | 0.477 | 0 | 1.047 | 0 | 0 | 0.579 | 0 | 0 | 0 | 0 | 0 | 0 | 0.325 | 0 | 0 | 0 |  | -0.95 |
| GAD3 | 0 | 1.047 | 0 | 1.084 | 0 | 0 | 0.468 | 0 | 0 | 0.293 | 0 | 0 | 1.124 | 0 | 0 | 0 |  | -2.11 |
| GAD4 | 0 | 0 | 1.084 | 0 | 0.931 | 0 | 0.687 | 0 | 0 | 0.322 | 0 | 0 | 0.255 | 0 | 0 | 0 |  | -1.17 |
| GAD5 | -0.362 | 0 | 0 | 0.931 | 0 | 2.003 | 0.482 | 0 | 0 | 0 | 0 | 0 | 0 | 0 | 0 | 0 |  | -1.41 |
| GAD6 | 0.720 | 0.579 | 0 | 0 | 2.003 | 0 | 1.155 | 0 | 0 | 0 | 0 | 0 | 0 | 0 | 0 | 0 |  | -2.46 |
| GAD7 | 2.103 | 0 | 0.468 | 0.687 | 0.482 | 1.155 | 0 | 0 | 0 | 0 | 0 | 0 | 0 | 0 | 0.242 | 0.685 |  | -3.30 |
| **Depression** |  |  |  |  |  |  |  |  |  |  |  |  |  |  |  |  |  |  |
| PHQ1 | 0 | 0 | 0 | 0 | 0 | 0 | 0 | 0 | 1.740 | 1.524 | 1.304 | 1.695 | 0 | 0 | 0 | 0 |  | -1.23 |
| PHQ2 | 0 | 0 | 0 | 0 | 0 | 0 | 0 | 1.740 | 0 | 1.099 | 1.061 | 1.919 | 0 | 0 | 0 | -0.928 |  | -1.61 |
| PHQ3 | 0 | 0 | 0.293 | 0.322 | 0 | 0 | 0 | 1.524 | 1.099 | 0 | 0 | -0.493 | 1.737 | 0.570 | 0 | 0 |  | -4.47 |
| PHQ4 | 0.294 | 0 | 0 | 0 | 0 | 0 | 0 | 1.304 | 1.061 | 0 | 0 | 1.514 | 3.072 | 0 | -2.882 | 1.695 |  | -2.77 |
| PHQ5 | 0 | 0 | 0 | 0 | 0 | 0 | 0 | 1.695 | 1.919 | -0.493 | 1.514 | 0 | 0 | 0.798 | 1.927 | 0 |  | -4.07 |
| PHQ6 | 0 | 0.325 | 1.124 | 0.255 | 0 | 0 | 0 | 0 | 0 | 1.737 | 3.072 | 0 | 0 | 0 | 1.925 | 0.748 |  | -5.38 |
| PHQ7 | 0 | 0 | 0 | 0 | 0 | 0 | 0 | 0 | 0 | 0.570 | 0 | 0.798 | 0 | 0 | 3.085 | 0 |  | -3.04 |
| PHQ8 | 0 | 0 | 0 | 0 | 0 | 0 | 0.242 | 0 | 0 | 0 | -2.882 | 1.927 | 1.925 | 3.085 | 0 | 1.773 |  | -3.52 |
| PHQ9 | 0.308 | 0 | 0 | 0 | 0 | 0 | 0.685 | 0 | -0.928 | 0 | 1.695 | 0 | 0.748 | 0 | 1.773 | 0 |  | -3.50 |

**Figures**





**Figure S1.** **Partial Correlation Network of Anxiety and Depression Based on The Original Continuous Scores for Each Symptom.**

This network is highly correlated with the Ising network estimated based on the binary scores for Each Symptom (*r* = .78, *p* = .001), with no significant differences.


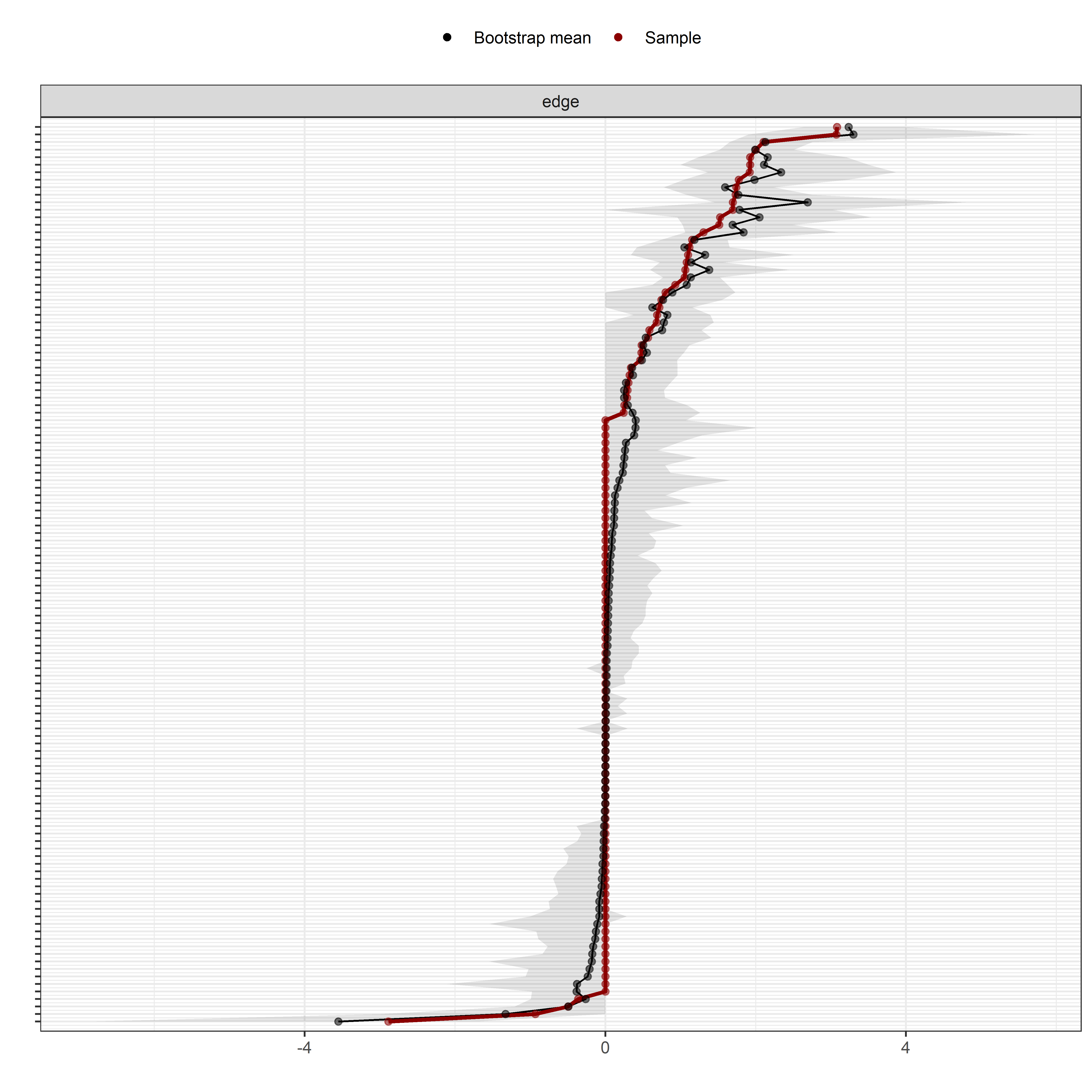


**Figure S2.** **Bootstrapped Accuracy Test for Confidence Intervals of Edges in The Network.**


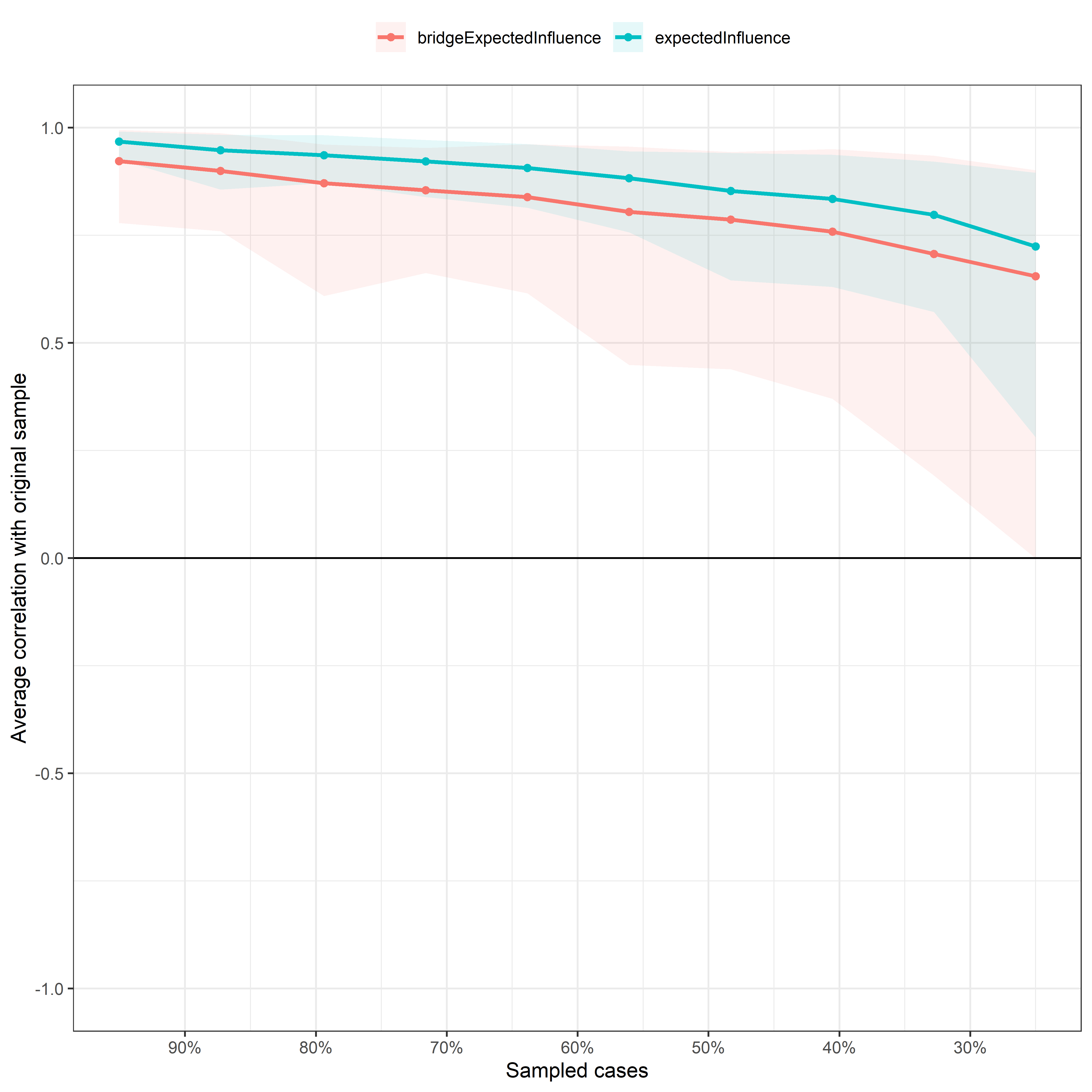


**Figure S3.** **Bootstrapped Stability Test for Node Centrality in The Network.**


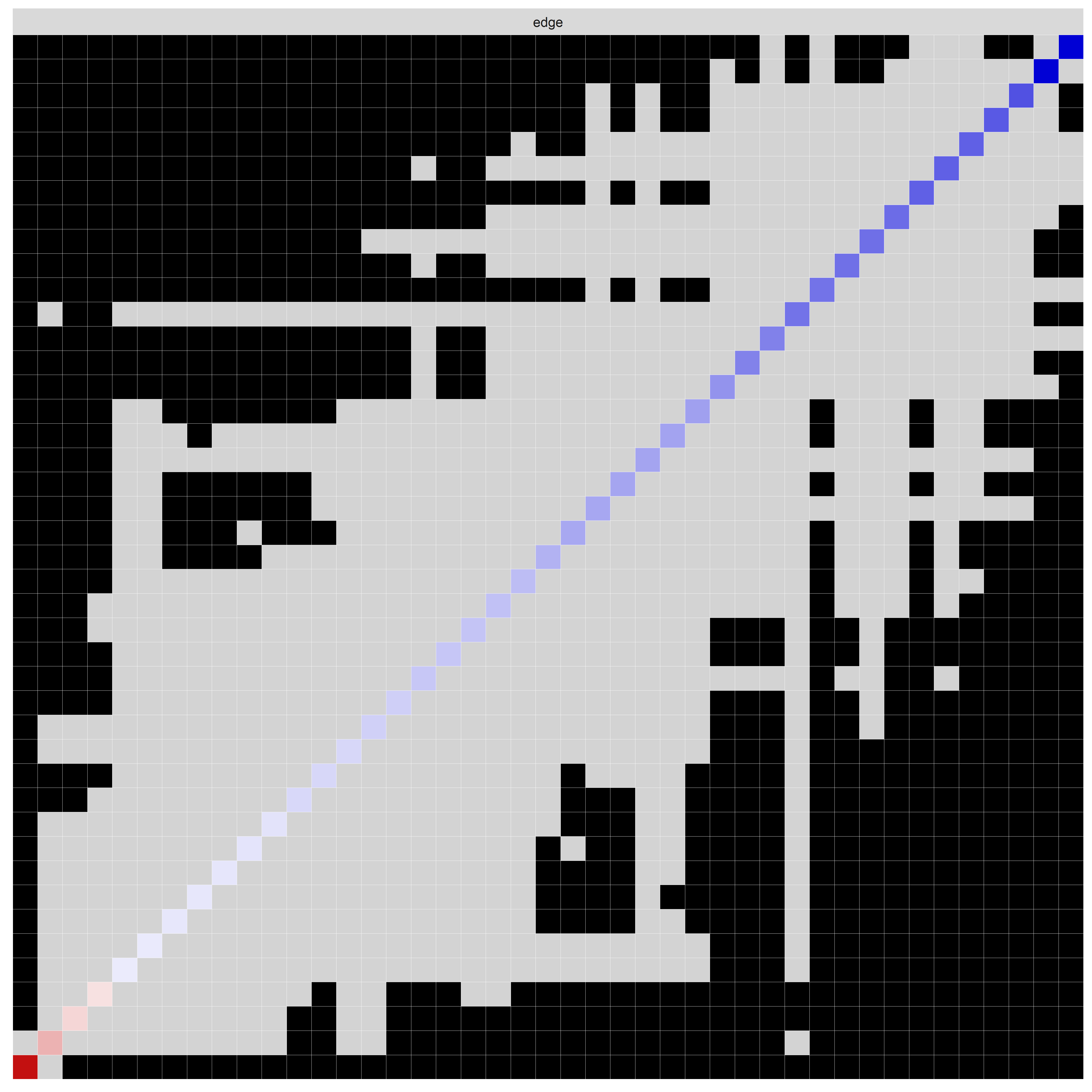


**Figure S4: Bootstrapped Difference Test for Edge-Weights in The Network.**

The color of the boxes represents whether the edge-weights differ significantly from each other (i.e., black) or do not differ significantly (i.e., grey). The diagonal line represents the strength of edge weights, changing from red (indicating negative associations) to white (representing weaker edges), and finally to blue (indicating stronger edge weights).


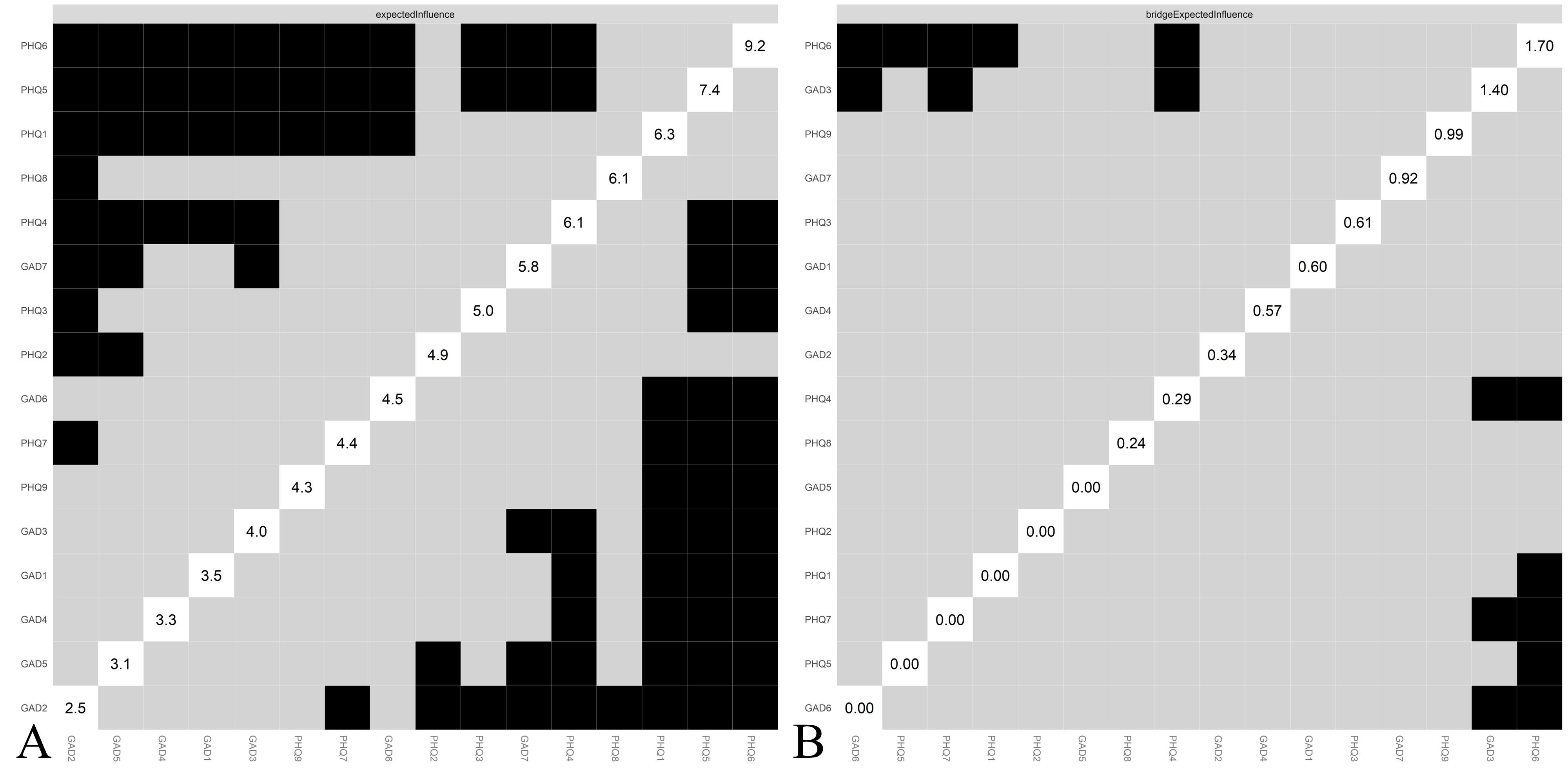


**Figure S5: Bootstrapped Difference Test for Node EI (A) and Bridge EI (B) Centrality in The Network.**

The color of the boxes represents whether the node centrality differ significantly from each other (i.e., black) or do not differ significantly (i.e., grey). The number in the white boxes (i.e., the diagonal line) denotes the value of the node strength of a specific node.
